# Early clinical determinants of adverse outcomes in paediatric febrile neutropenia: a prospective cohort study

**DOI:** 10.3389/fonc.2026.1876009

**Published:** 2026-07-29

**Authors:** Manideepa Maji, Nita Radhakrishnan, Shruti Verma, Aishvarya Diptivilasa, Saikat Mandal

**Affiliations:** 1 Postgraduate Institute of Child Health, Noida, India; 2 Hull York Medical School, University of Hull, Hull, United Kingdom; 3 Department of Haematology, Hull University Teaching Hospitals NHS Trust, Hull, United Kingdom; 4 Translational Medical Sciences, School of Medicine, University of Nottingham, Nottingham, United Kingdom; 5 NIHR Nottingham Biomedical Research Centre, Nottingham University Hospitals NHS Trust, Nottingham, United Kingdom

**Keywords:** blood cancer, childhood cancer, febrile neutropenia (FN), neutropenic fever, paediatric cancer, paediatric cancer care

## Abstract

**Background:**

Febrile neutropenia (FN) remains a major cause of morbidity and mortality in paediatric haemato-oncology, but routinely available determinants of adverse in-hospital outcome remain incompletely characterised. We aimed to identify baseline clinical, haematological, biochemical, and microbiological factors associated with in-hospital mortality and prolonged fever.

**Methods:**

We conducted a prospective single-centre cohort study of 90 consecutive FN admissions in paediatric haemato-oncology patients. Thirty-nine prespecified baseline predictors were evaluated using Firth penalised-likelihood logistic regression. Multivariable models were prespecified on clinical grounds, and discrimination was internally validated using 500 bootstrap replicates with optimism correction. Prolonged fever was defined as time to defervescence >5 days, with death before defervescence counted as an event.

**Results:**

Of 86 mortality-evaluable episodes, 16 children died in hospital (18.6%). In the multivariable mortality model, any respiratory support (adjusted odds ratio [aOR], 11.9; 95% CI, 2.5–56.9) and lower admission albumin (aOR, 0.29 per 1 g/dL increase; 95% CI, 0.11–0.77) showed the strongest associations with death. Active disease (aOR 3.5, 95% CI 0.87–14.2) and gram-negative bacteraemia (aOR 4.2, 95% CI 0.93–18.9) showed directionally adverse but imprecise associations. The bootstrap-corrected AUC was 0.88. Of 60 classifiable episodes for the secondary outcome, 31 (51.7%) had prolonged fever. Prolonged fever was associated with older age (aOR 1.24 per year, 95% CI 1.05–1.48), lower albumin (aOR 0.31 per 1 g/dL increase, 95% CI 0.12–0.82), any respiratory support (aOR 10.0, 95% CI 0.98–102.0), and gram-negative bacteraemia (aOR 5.5, 95% CI 1.01–30.3). The bootstrap-corrected AUC was 0.85. Neutropenia depth and platelet count were not independently associated with either outcome.

**Conclusion:**

In paediatric haemato-oncology patients with FN, adverse in-hospital outcomes were associated mainly with early or evolving respiratory compromise, lower admission albumin, active disease, and gram-negative bacteraemia. Three of these factors, together with age, were associated with prolonged fever or death before defervescence among classifiable episodes. Albumin should be interpreted as a dynamic marker of acute inflammatory burden, capillary leak, and host reserve rather than as a standalone nutritional measure. These routinely available variables may support exploratory bedside risk appraisal, but external validation is required before clinical implementation.

## Introduction

Febrile neutropenia (FN) remains one of the most time-critical complications of anticancer therapy in children and is a major cause of morbidity, intensive care admission, treatment interruption, and death in paediatric haematology-oncology practice (1–5). Although prompt empirical antibiotic therapy has substantially improved outcomes, children with cancer or marrow failure syndromes remain uniquely vulnerable because infection risk is shaped not only by neutropenia but also by underlying diagnosis, disease status, treatment intensity, mucosal barrier injury, previous antimicrobial exposure, central venous access, and local pathogen epidemiology (6). This is particularly relevant in mixed paediatric haemato-oncology populations, in which children with acute lymphoblastic leukaemia, acute myeloid leukaemia, aplastic anaemia, lymphoma, and solid tumours differ substantially in baseline risk and in the mechanisms by which infection progresses to organ dysfunction.

Current guidance increasingly emphasises that adverse outcomes in paediatric infection are determined by early physiological compromise rather than by fever or cytopenia alone (2, 7, 8). The 2024 Phoenix consensus redefined paediatric sepsis as infection-associated life-threatening organ dysfunction and moved practice away from older SIRS-based concepts (7). Likewise, the 2026 Surviving Sepsis Campaign paediatric guidelines emphasise early recognition of sepsis and septic shock, rapid antimicrobials within recommended timeframes, careful haemodynamic reassessment, judicious fluid and vasoactive support, and timely escalation of organ support (8). In paediatric oncology, contemporary fever and neutropenia guidance from the Children’s Oncology Group and related supportive-care groups recommends immediate risk assessment, blood culture acquisition, and early empirical anti-pseudomonal therapy for high-risk episodes, while recommendations from the European Conference on Infections in Leukaemia (ECIL) similarly support stratified escalation according to clinical instability, resistant-pathogen risk, and microbiological context (2, 9). Persistent fever beyond the first several days of therapy also has direct management implications, as it triggers reassessment for occult bacterial or invasive fungal infection and may influence imaging, antifungal escalation, and the duration of admission (2, 10).

Despite these advances, an important gap remains. Most available paediatric FN studies focus either on broad composite outcomes or on narrowly defined subgroups, and relatively few examine how routinely available variables determine short-term outcomes across children with different haemato-oncology diagnoses in real-world inpatient practice (11). It remains uncertain whether diagnosis category itself, markers of disease biology, bedside physiological variables, microbiological findings, or laboratory markers of host reserve are the most informative determinants of sepsis-related outcome once a child presents to the hospital (12, 13). Clarifying this is clinically important because early identification of children at the highest risk of death or prolonged complicated fever could improve monitoring intensity, support earlier escalation to intensive care, guide the empirical breadth of antimicrobials, and support more personalised supportive care (14–16).

We therefore analysed consecutive admissions for FN in paediatric haematology-oncology patients during 2021–2022 to determine how baseline demographic, disease-related, physiological, biochemical, haematological, and microbiological variables were associated with two clinically relevant in-hospital outcomes: mortality and prolonged fever. Our primary research question was whether routinely available variables could identify adverse neutropenic-fever outcomes in paediatric haemato-oncology patients across different underlying diagnoses, and whether a small set of these factors could identify a shared severity axis while also highlighting outcome-specific predictors.

## Methods

### Study design and participants

We conducted a prospective cohort study of consecutive admissions with febrile neutropenia among paediatric haematology-oncology patients at a single tertiary paediatric centre, Postgraduate Institute of Child Health, Noida, India. We prospectively screened all consecutive admissions for eligibility at the time of presentation and enrolled eligible febrile neutropenia episodes during the study period. Baseline clinical, laboratory, and microbiological variables were defined *a priori* and recorded at admission or during the early clinical assessment, before study outcomes were assigned. Eligible episodes were in patients aged less than 18 years with an underlying haematological or solid malignancy or with aplastic anaemia, presenting with neutropenic fever during or within the expected window of chemotherapy-induced neutropenia. For the study purpose, febrile neutropenia was defined as a single oral temperature measurement of ≥38.3 °C (≥101°F) or a temperature of ≥38.0 °C (≥100.4°F) sustained over 1 hour, with an absolute neutrophil count (ANC) of ≤500 cells/µL, or an ANC of ≤1000 cells/µL expected to decrease to ≤500 cells/µL over the next 48 hours (1). The study was conducted in accordance with institutional ethical review.

### Outcomes

The primary outcome was in-hospital mortality, coded from the attending physician’s disposition field (discharge vs death). Episodes without an ascertainable final in-hospital vital status were treated as missing for the primary outcome. The secondary outcome was prolonged fever, defined *a priori* as time from fever onset to defervescence greater than 5 days, or death before defervescence. Episodes without a documented defervescence date were considered unclassifiable for this outcome and were excluded from the primary prolonged-fever analysis. We did not infer defervescence from discharge status alone because the exact timing of fever resolution relative to the prespecified >5-day threshold could not be verified. The 5-day threshold aligns with consensus recommendations at which empirical antifungal escalation and investigation for occult infection are considered in persistent paediatric febrile neutropenia (2).

### Data collection and preparation

Data were collected prospectively using a structured case-record form and were subsequently cross-checked against the hospital record for completeness and accuracy before analysis. Outcome definitions and candidate predictors were prespecified in the study protocol before statistical modelling. Following prospective enrolment, baseline variables were recorded at presentation or admission and verified from the patient’s health record available at the Postgraduate Institute of Child Health, Noida, India; the final dataset was then entered into a single Excel analysis file. Community-onset vs hospital-acquired febrile neutropenia was determined by the interval between the fever onset date, as mentioned in the patient’s medical history, and the admission date. Respiratory-support variables captured whether oxygen, high-flow nasal cannula, or mechanical ventilation was required during the early inpatient course; because exact initiation timestamps were not available in the current dataset, these variables were interpreted as markers of early or evolving organ dysfunction rather than fixed pre-admission exposures.

### Candidate predictors

Thirty-nine baseline predictors were pre-specified for screening across eight domains: (a) demographics (age, sex, body mass index); (b) disease characteristics (diagnosis category, active disease [defined as refractory, minimal residual disease-positive, or disease-positive status], relapsed/refractory in diagnosis string, intensive-phase chemotherapy, community-onset vs hospital-acquired FN); (c) vital signs at presentation (heart rate, respiratory rate, systolic and diastolic blood pressure, age-adjusted hypotension, peripheral oxygen saturation, Glasgow Coma Scale, urine output); (d) respiratory support (supplemental oxygen, high-flow nasal cannula, mechanical ventilation, and a composite “any respiratory support”); (e) supportive interventions (fluid resuscitation, significant oedema); (f) haematology (total leukocyte count, absolute neutrophil count [continuous and binary thresholds at <100, <500, <1000/µL], platelet count [continuous and <50 × 10³/µL binary]); (g) biochemistry and microbiology (creatinine, age-adjusted acute kidney injury, albumin, hypoalbuminaemia <3.5 g/dL, sodium, potassium, random blood glucose, immunoglobulin G [IgG], any positive blood culture, gram-negative bacteraemia); and (h) prior exposures (corticosteroids). Antibiotic and supportive-care regimens were excluded from the prognostic model because these are administered in response to severity and, therefore, are vulnerable to reverse causality.

### Statistical analysis

Baseline characteristics were compared between survivors and non-survivors by the Mann-Whitney U test for continuous variables and by Fisher’s exact test (or chi-square test for variables with more than two categories) for categorical variables. Each candidate predictor was then fitted in univariable logistic regression against each outcome using Firth penalised-likelihood estimation throughout, applied uniformly to all univariable and multivariable models. Firth’s penalty reduces small-sample and separation-induced bias and yields finite estimates when complete or quasi-complete separation occurs. Because Wald and likelihood-ratio inferences are not algebraically equivalent under penalised estimation, small discrepancies between confidence-interval bounds and per-coefficient P values are expected. For the univariable screen, Benjamini-Hochberg false-discovery-rate (FDR) q values were calculated separately for the mortality and prolonged-fever outcomes across the 39 *a priori* predictors; the *post hoc* severe-thrombocytopenia threshold was displayed but excluded from the FDR family. Univariable screening was considered exploratory, and multivariable models were not selected by univariable P values. For graphical presentation, univariable predictors were ranked by P-value, and the strongest associations are displayed in forest plots.

Multivariable models were pre-specified on clinical grounds in accordance with recommendations for prognostic modelling with low event counts. Predictors were chosen to represent four distinct mechanistic axes of severity rather than by univariable significance, ensuring that each coefficient captures a non-overlapping component of risk and avoiding the model instability that arises from including correlated predictors when events are limited. For the primary outcome (16 deaths), the pre-specified four-predictor model combined acute physiological decompensation (any respiratory support), chronic nutritional-inflammatory state (albumin, modelled continuously per 1 g/dL), disease status (active disease), and causative microbiology (gram-negative bacteraemia). This gave an events-per-variable ratio of 4.0. For the secondary outcome (31 events), the pre-specified four-predictor model included age, albumin (continuous), any respiratory support, and gram-negative bacteraemia, with an events-per-variable ratio of approximately 7.8. These values fall below the conventional EPV≥10 rule of thumb and are the explicit rationale for penalised estimation and cautious interpretation. Model discrimination was summarised by the area under the receiver operating characteristic curve (AUC). Internal validation used 500 bootstrap replicates with optimism correction, where optimism was defined as the mean difference between the in-bag AUC of the bootstrap-fitted model and its performance on the original sample; the corrected AUC was calculated as the apparent AUC minus optimism. The AUCs are reported as internal discrimination only and not as evidence of ready clinical deployment. An exploratory *post hoc* analysis examined lower thresholds of thrombocytopenia (<10 × 10³/µL), given that the conventional <50 × 10³/µL threshold was met by 84% of the cohort and therefore was unable to stratify risk; this analysis was not pre-specified and is reported as hypothesis-generating.

Two sensitivity analyses were performed. First, the primary mortality model was refitted using only the first episode per patient to address within-patient clustering in 10 patients with repeat admissions. Second, an L1-regularised (Lasso) logistic regression with nested cross-validation (10 repeats × 5 outer folds, 5 inner folds for hyperparameter tuning) was used as a data-driven sensitivity analysis of variable selection; variables selected in at least 80% of outer folds were considered stable. Continuous time to defervescence was additionally analysed with death as a competing event using Aalen-Johansen cumulative incidence and cause-specific Cox regression, as an exploratory complement to the binary prolonged-fever outcome.

Missing data were handled as follows: continuous variables with isolated missingness (random blood glucose, 1 case; diastolic blood pressure, 1 case) were median imputed for multivariable models. IgG was missing in 16 of 86 episodes (19%), with missingness strongly associated with outcome (56% in deaths vs 10% in survivors); complete-case, median-imputed, and multiple-imputation (m = 20 using iterative random-forest imputation) analyses all gave null estimates for IgG effect, and IgG was therefore not retained in the multivariable model. For prolonged fever, 60 of 90 episodes were classifiable; non-classifiable episodes were described and compared with classifiable episodes, and best- and worst-case bounds were calculated by assigning all non-classifiable episodes as non-prolonged and prolonged, respectively. All analyses were two-sided with α = 0.05. The significance threshold was interpreted as a continuous strength-of-evidence metric rather than a dichotomous decision rule. Analyses were performed in Python 3.12 using statsmodels 0.14, scipy, lifelines 0.30, and scikit-learn 1.4, with a local implementation of Firth penalised logistic regression. All analyses were independently replicated in R 4.5.1 using the logistf package (version 1.26.1); point estimates and bootstrap-corrected AUCs matched the primary Python analysis.

## Results

### Cohort characteristics

Ninety febrile neutropenia episodes occurred in 78 patients between December 2021 and December 2022; 86 episodes had definitive in-hospital disposition (70 discharge, 16 death) and formed the primary analysis set, whereas 3 episodes ending in Left Against Medical Advice (LAMA) and 1 in Discharge on Patient Request (DOPR) were excluded because final in-hospital vital status could not be reliably ascertained. Median age was 8 years (IQR, 4–12), and 61 (67.8%) patients were male. Acute lymphoblastic leukaemia/lymphoid leukaemia accounted for 61 of 90 (67.8%) episodes, followed by acute myeloid leukaemia (12 of 90, 13.3%), aplastic anaemia (8 of 90, 8.9%), lymphoma (4 of 90, 4.4%), solid tumours (4 of 90, 4.4%), and Langerhans cell histiocytosis (1 of 90, 1.1%). Active disease at fever onset (refractory, MRD-positive, or disease-positive) was present in 26.7% of episodes. Community-onset febrile neutropenia represented 85.6% of admissions. Severe cytopenia was near-universal: 51 of 90 (56.7%) had ANC <100/µL, and 76 of 90 (84.4%) had platelets <50 × 10³/µL. Blood cultures were positive in 19 of 90 (21.1%), predominantly gram-negative organisms. In-hospital mortality was 16/86 (18.6%). Baseline characteristics are presented in **Table 1**.

**Table 1:** Baseline characteristics of the study cohort, by in-hospital outcome.

Characteristic	Survived (n=70)	Died (n=16)	P
Demographics			
Age (year), median (IQR)	8.0 (4.0–12.0)	8.5 (7.0–12.0)	0.22
Male sex, No. (%)	53 (75.7)	8 (50.0)	0.07
BMI, kg/m², median (IQR)	14.8 (13.7–15.8)	14.8 (13.7–16.2)	0.89
Disease			
Underlying diagnosis, No. (%)			0.07
ALL/lymphoid leukaemia	52 (74.3)	8 (50.0)	
AML	7 (10.0)	3 (18.8)	
Aplastic anaemia	3 (4.3)	4 (25.0)	
Lymphoma/other	8 (11.5)	1 (6.2)	
Active disease*, No. (%)	14 (20.0)	9 (56.2)	0.009
Intensive-phase chemotherapy^†^, No. (%)	40 (59.7)	9 (75.0)	0.52
Community-onset FN, No. (%)	63 (90.0)	14 (87.5)	0.67
Vital signs and severity			
Heart rate, bpm	126.5 (112.0–143.0)	145.0 (142.8–165.0)	<0.001
Respiratory rate,/min	26.5 (24.2–32.0)	32.0 (26.5–35.0)	0.05
Systolic BP, mmHg	100.0 (96.0–107.8)	97.0 (90.8–98.5)	0.03
Diastolic BP, mmHg	65.0 (56.0–69.0)	48.0 (45.0–56.0)	<0.001
SpO_2_, %	99.0 (98.0–99.8)	98.0 (95.0–99.0)	0.007
Urine output, mL/kg/h	3.7 (2.5–4.3)	3.0 (1.2–3.9)	0.12
Any respiratory support, No. (%)	4 (5.7)	8 (50.0)	<0.001
Supplemental O_2_	4 (5.7)	8 (50.0)	<0.001
High-flow nasal cannula	1 (1.4)	7 (43.8)	<0.001
Mechanical ventilation	0 (0.0)	7 (43.8)	<0.001
Fluid resuscitation, No. (%)	3 (4.3)	4 (25.0)	0.02
Significant oedema, No. (%)	2 (2.9)	8 (50.0)	<0.001
Complete blood count			
TLC, cells/µL	981 (525–1855)	779 (168–1728)	0.36
ANC, cells/µL	84 (22–280)	16 (2.0–343.0)	0.14
ANC <100/µL, No. (%)	38 (54.3)	10 (62.5)	0.59
Platelets, ×10³/µL	18.2 (9.2–36.2)	6.3 (2.6–46.1)	0.15
Platelets <50 ×10³/µL, No. (%)	59 (84.3)	13 (81.2)	0.72
Biochemistry			
Creatinine, mg/dL	0.4 (0.3–0.5)	0.6 (0.5–0.6)	0.008
Albumin, g/dL	3.5 (2.9–3.9)	2.6 (2.2–3.1)	<0.001
Hypoalbuminemia <3.5 g/dL, No. (%)	32 (45.7)	13 (81.2)	0.01
Sodium, mmol/L	132.0 (129.2–134.0)	129.5 (127.5–132.2)	0.21
Potassium, mmol/L	3.7 (3.2–3.9)	3.2 (2.9–3.7)	0.17
Random blood glucose, mg/dL	118.0 (102.0–143.0)	106.5 (88.5–125.5)	0.11
IgG, mg/dL (measured cases)	728 (558–886)	769 (507–776)	0.48
Microbiology			
Any positive blood culture, No. (%)	12 (17.1)	6 (37.5)	0.09
Gram-negative bacteraemia, No. (%)	11 (15.7)	6 (37.5)	0.08

ALL, acute lymphoblastic leukaemia; AML, acute myeloid leukaemia; ANC, absolute neutrophil count; BMI, body mass index; BP, blood pressure; FN, febrile neutropenia; IgG, immunoglobulin G; IQR, interquartile range; SpO_2_, peripheral oxygen saturation; TLC, total leukocyte count. Continuous variables: median (IQR), compared by Mann-Whitney U test. Categorical variables: No. (%), compared by Fisher’s exact test. *Refractory, MRD-positive, or disease-positive status at FN onset. ^†^Induction, reinduction, high-risk cycle, HIDAC, COPADM, or salvage regimens vs consolidation, maintenance, or other.

### Univariable analysis: in-hospital mortality

**Figure 1:**
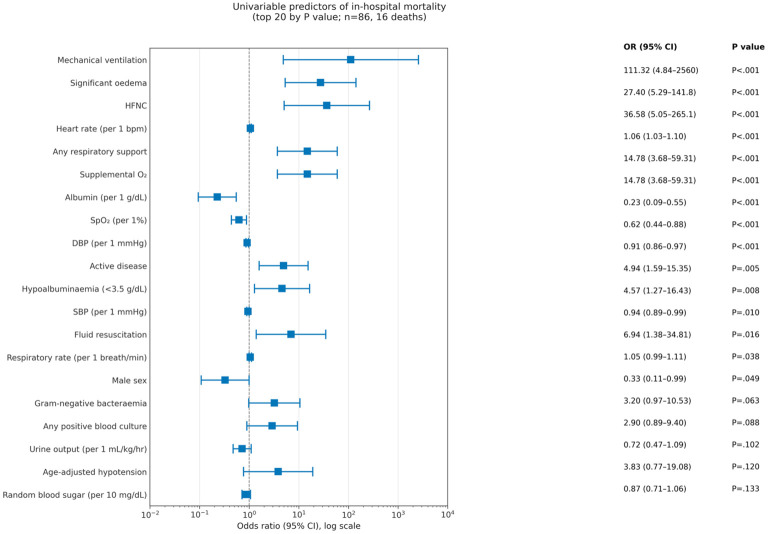
Univariable predictors of in-hospital mortality in paediatric febrile neutropenia (n = 86, 16 deaths). The forest plot shows the top 20 predictors, sorted by P-value. Odds ratios are displayed on a logarithmic scale with 95% confidence intervals. All estimates are derived from Firth penalised-likelihood logistic regression.

All 40 candidate predictors (39 pre-specified and one *post hoc* threshold) were screened against in-hospital mortality (**Figure 1**; **Supplementary Table 1**). The strongest univariable associations were markers of acute physiological decompensation and end-organ dysfunction. Among ventilated patients, none survived (0 of 7); the corresponding Firth penalised estimate was OR 111.32 (95% CI, 4.84 to 2560; P < 0.001). High-flow nasal cannula (OR 36.58; 95% CI, 5.05 to 265.11; P < 0.001), significant oedema (OR 27.40; 95% CI, 5.29 to 141.79; P < 0.001), and the composite any respiratory support (OR 14.78; 95% CI, 3.68 to 59.31; P < 0.001) were similarly strong. Continuous physiological variables were also significant: heart rate per bpm (OR 1.06; 95% CI, 1.03 to 1.10; P < 0.001), albumin per g/dL (OR 0.23; 95% CI, 0.09 to 0.55; P < 0.001), peripheral oxygen saturation per percentage point (OR 0.62; 95% CI, 0.44 to 0.88; P < 0.001), and diastolic blood pressure per mmHg (OR 0.91; 95% CI, 0.86 to 0.97; P < 0.001). Active disease (OR 4.94; 95% CI, 1.59 to 15.35; P = 0.005), hypoalbuminaemia at the conventional <3.5 g/dL threshold (OR 4.57; 95% CI, 1.27 to 16.43; P = 0.008), fluid resuscitation (OR 6.94; 95% CI, 1.38 to 34.81; P = 0.016), and gram-negative bacteraemia (OR 3.20; 95% CI, 0.97 to 10.53; P = 0.063) were also associated. After Benjamini-Hochberg correction, 13 predictors retained q<0.05 for mortality, including respiratory-support markers, oedema, low albumin, active disease, hypoalbuminaemia, systolic and diastolic blood pressure, heart rate, SpO_2_, and fluid resuscitation (**Supplementary Table 1a**). Male sex showed lower odds of death before FDR correction (OR 0.33; 95% CI, 0.11 to 0.99; P = 0.049; q = 0.128).

No haematological parameter reached statistical significance or survived FDR correction: total leukocyte count (P = 1.00; q = 1.00), absolute neutrophil count as a continuous variable (P = 0.87; q = 0.964) or at thresholds of <100/µL, <500/µL, or <1000/µL, and platelet count as continuous or <50×10³/µL were all null. An exploratory *post hoc* analysis identified severe thrombocytopenia (<10×10³/µL) as a univariable predictor of mortality (OR 3.12; 95% CI, 1.04 to 9.38; P = 0.040; observed in 29 of 86 episodes; **Supplementary Table 1**), but this threshold was not part of the prespecified FDR family. IgG (where measured; P = 0.244; q = 0.396), body mass index, random blood glucose, and intensive-phase chemotherapy were similarly not significant. Full estimates are reported in **Supplementary Table 1**, with FDR q values in **Supplementary Table 1a**.

### Multivariable analysis: in-hospital mortality

**Figure 2:**
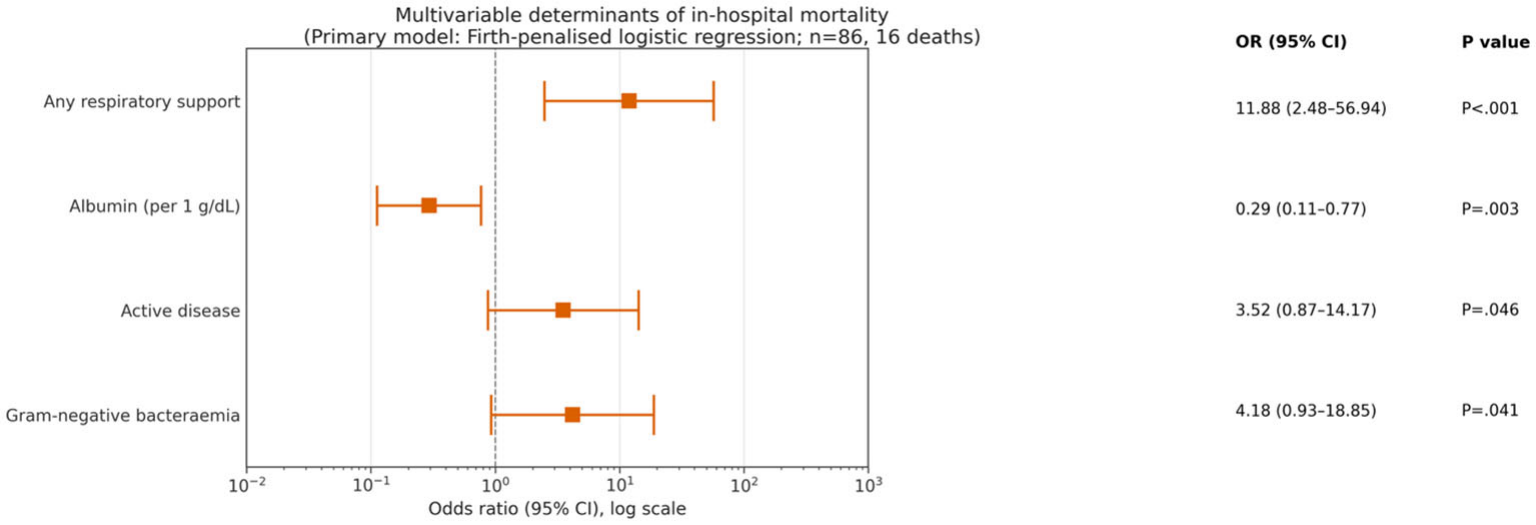
Adjusted odds ratios for in-hospital mortality from the primary multivariable model. Firth penalised-likelihood logistic regression; 95% confidence intervals shown on logarithmic scale.

**Figure 3:**
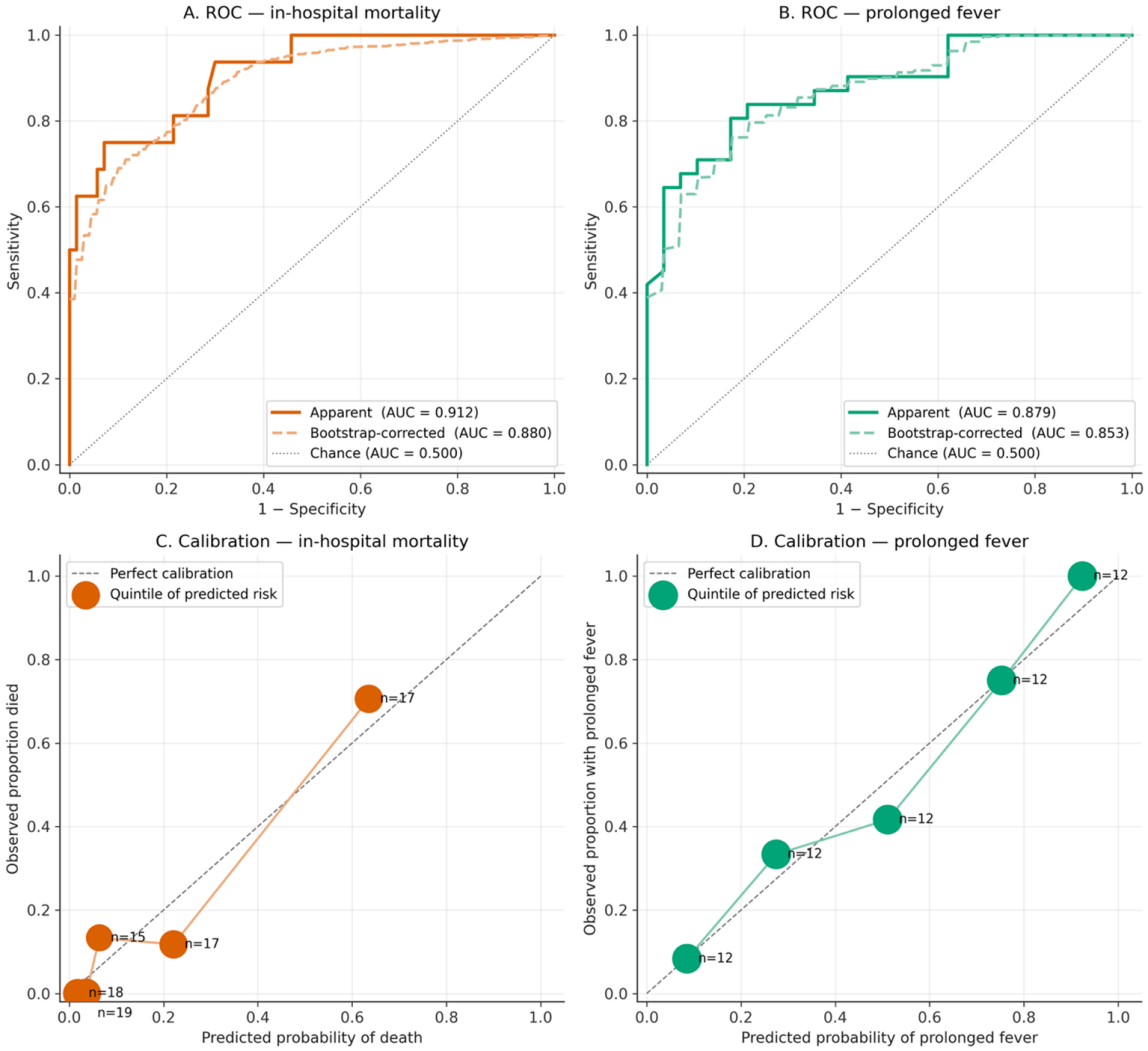
Model discrimination and calibration. **(A)** Receiver operating characteristic (ROC) curves for the primary multivariable model of in-hospital mortality. The solid line shows apparent discrimination in the derivation sample (apparent AUC 0.912); the dashed line shows bootstrap-optimism-corrected discrimination (corrected AUC 0.880) after 500 bootstrap replicates. **(B)** ROC curves for the secondary multivariable model of prolonged fever (defined as time to defervescence > 5 days or death before defervescence); apparent AUC 0.879, bootstrap-corrected AUC 0.853. **(C)** Calibration plot for the mortality model. Points represent quintiles of predicted risk; marker size is proportional to the number of patients per bin; the dashed line represents perfect calibration. **(D)** Calibration plot for the prolonged-fever model, constructed identically.

The pre-specified four-predictor multivariable model is presented in **Table 2** and **Figure 2**. Any respiratory support showed the largest association with in-hospital mortality (adjusted OR, 11.88; 95% CI, 2.48 to 56.94; P < 0.001). Each 1 g/dL increase in admission albumin was associated with approximately 71% lower odds of death (adjusted OR, 0.29; 95% CI, 0.11 to 0.77; P = 0.003). Active disease (adjusted OR, 3.52; 95% CI, 0.87 to 14.17; P = 0.046) and gram-negative bacteraemia (adjusted OR, 4.18; 95% CI, 0.93 to 18.85; P = 0.041) showed directionally adverse but imprecise associations; their Wald-based confidence intervals included or marginally crossed unity, reflecting the limited number of events. Model penalised likelihood-ratio χ²(4) = 33.9 (P < 0.001); apparent AUC was 0.912, and bootstrap-optimism-corrected AUC was 0.880. Calibration across quintiles of predicted risk was preserved (**Figure 3**).

**Table 2:** Multivariable predictors of in-hospital mortality in paediatric febrile neutropenia.

Predictor	Adjusted OR (95% CI)	P
Any respiratory support	11.88 (2.48–56.94)	<0.001
Albumin (per 1 g/dL)	0.29 (0.11–0.77)	0.003
Active disease	3.52 (0.87–14.17)	0.046
Gram-negative bacteraemia	4.18 (0.93–18.85)	0.041

AUC, area under the receiver operating characteristic curve; CI, confidence interval. Firth penalised-likelihood logistic regression. N = 86 episodes, 16 deaths. Likelihood ratio χ²(4) = 33.9, P < 0.001. Apparent AUC = 0.912; bootstrap-optimism-corrected AUC = 0.880 (500 iterations). Albumin is modelled as a continuous variable; the OR represents the change in odds of in-hospital mortality per 1 g/dL increase in admission albumin. Confidence intervals are Wald-type while P values come from the penalised likelihood-ratio test.

### Sensitivity analyses

Restriction of the primary mortality model to one episode per patient (the death admission for the one patient who died during a later episode, otherwise the first admission) gave 74 evaluable episodes with 16 deaths. Refitting the same four-predictor Firth model with continuous albumin, matching the primary parameterisation, produced directionally and quantitatively consistent estimates: any respiratory support adjusted OR, 12.25 (95% CI, 2.31 to 64.87; P < 0.001); albumin per 1 g/dL adjusted OR, 0.30 (95% CI, 0.11 to 0.79; P = 0.005); active disease adjusted OR, 2.97 (95% CI, 0.69 to 12.85; P = 0.143); and gram-negative bacteraemia adjusted OR, 4.52 (95% CI, 0.92 to 22.25; P = 0.050). Apparent AUC was 0.914 and bootstrap-optimism-corrected AUC was 0.884. Effect direction and approximate magnitude were preserved, suggesting that the primary findings were not an artefact of within-patient clustering (**Supplementary Table 6**).

An L1-regularised (Lasso) logistic regression with nested cross-validation (10 repeats × 5 outer folds, 5 inner folds for selection of the L1 penalty) over all 39 *a priori* candidate predictors reinforced the importance of physiological severity markers and active disease. Three predictors were selected in 100% of outer cross-validation folds (active disease, mechanical ventilation, and significant oedema), and heart rate was selected in 98% of folds. The mean out-of-fold AUC was 0.929 (SD 0.084 across 50 fold-level estimates from 10 × 5-fold cross-validation repeats). No haematological variable (TLC, ANC continuous or thresholded at <100, <500, or <1000/µL, or platelet count continuous or <50×10³/µL) was selected in more than 10% of folds, concordant with the null univariable and FDR findings for these parameters.

### Secondary outcome: prolonged fever

Of 90 episodes, 60 were classifiable for prolonged fever and 30 were not classifiable: 26 discharged episodes lacked a documented defervescence date, and 4 episodes ended in LAMA or discharge on patient request without reliable final in-hospital outcome ascertainment. These episodes were excluded from the primary prolonged-fever analysis, leaving 60 episodes, of which 31 (51.7%) had prolonged fever. Classifiable and non-classifiable episodes were similar across examined baseline characteristics, including age, BMI, albumin, active disease, any respiratory support, culture positivity, gram-negative bacteraemia, and intensive-phase chemotherapy (all P≥0.13; **Supplementary Table 2**). In sensitivity bounds, the prolonged-fever prevalence ranged from 31/90 (34.4%) if all non-classifiable episodes were assumed not prolonged to 61/90 (67.8%) if all were assumed prolonged.

### Univariable analysis: prolonged fever

**Figure 4:**
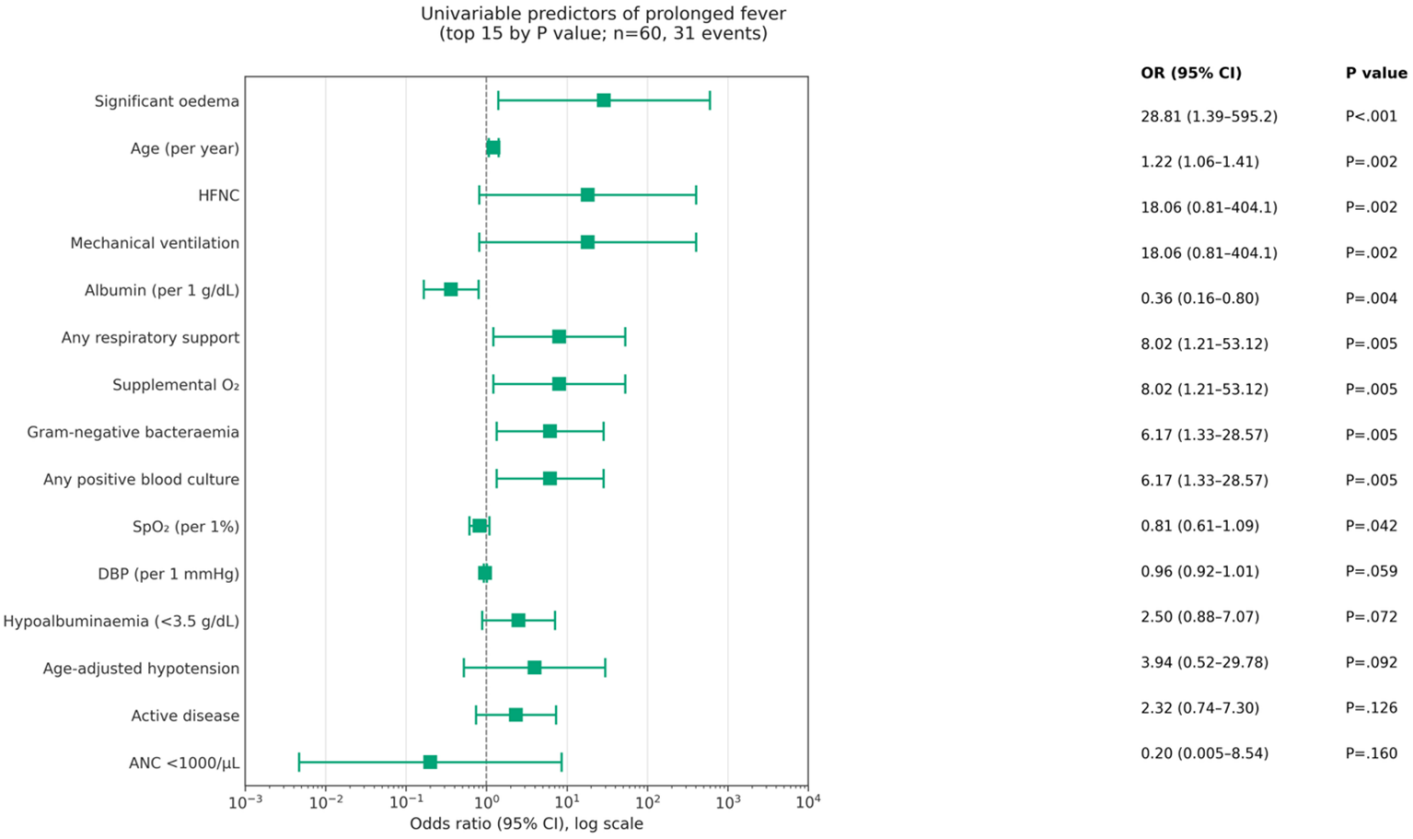
Univariable predictors of prolonged fever in paediatric febrile neutropenia (n = 60, 31 events). Forest plot shows the top 15 predictors sorted by *P* value. Odds ratios are displayed on a logarithmic scale with 95% confidence intervals. All estimates are derived from Firth penalised-likelihood logistic regression.

All 40 candidate predictors were screened against the secondary outcome (**Figure 4**; **Supplementary Table 1**). The strongest univariable association was significant oedema (OR 28.81; 95% CI, 1.39 to 595.20; P < 0.001; q = 0.007), although the very wide confidence interval reflects sparse cell counts at this stratification level. Markers of acute respiratory compromise were also strongly associated: high-flow nasal cannula and mechanical ventilation each yielded an OR of 18.06 (95% CI, 0.81 to 404.07; P = 0.002; q = 0.023), and any respiratory support showed an OR of 8.02 (95% CI, 1.21 to 53.12; P = 0.005; q = 0.023). Microbiological status was informative: gram-negative bacteraemia and any positive blood culture each yielded an OR of 6.17 (95% CI, 1.33 to 28.57; P = 0.005; q = 0.023). Among continuous markers, age increased the odds of prolonged fever by approximately 22% per year (OR 1.22; 95% CI, 1.06 to 1.41; P = 0.002; q = 0.023), and each 1 g/dL increase in albumin reduced the odds by approximately two-thirds (OR 0.36; 95% CI, 0.16 to 0.80; P = 0.004; q = 0.023). Hypoalbuminaemia, age-adjusted hypotension, low diastolic blood pressure, and SpO_2_ showed directionally consistent but less robust associations after FDR correction. As with mortality, no haematological parameter reached statistical significance after FDR correction. Active disease, which was retained in the mortality model, did not reach significance for prolonged fever (OR 2.32; 95% CI, 0.74 to 7.30; P = 0.126; q = 0.350).

### Multivariable analysis: prolonged fever

**Figure 5:**
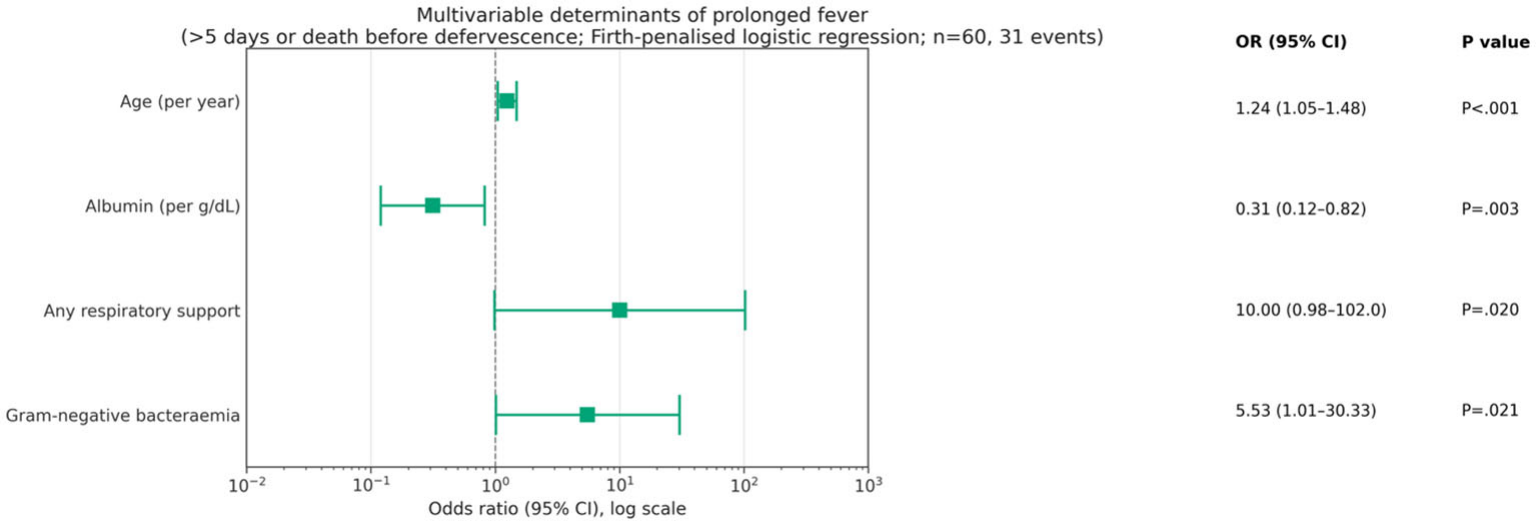
Adjusted odds ratios for prolonged fever from the multivariable model. Firth penalized-likelihood logistic regression. Prolonged fever was defined *a priori* as time to defervescence >5 days or death before defervescence.

The multivariable model is presented in **Table 3** and **Figure 5**. All four predictors showed directionally consistent associations with prolonged fever: age per year (adjusted OR, 1.24; 95% CI, 1.05 to 1.48; P < 0.001), albumin per g/dL (adjusted OR, 0.31; 95% CI, 0.12 to 0.82; P = 0.003), any respiratory support (adjusted OR, 10.00; 95% CI, 0.98 to 102.04; P = 0.020), and gram-negative bacteraemia (adjusted OR, 5.53; 95% CI, 1.01 to 30.33; P = 0.021). Model penalised likelihood-ratio χ²(4) = 31.2 (P < 0.001); apparent AUC was 0.879, and bootstrap-optimism-corrected AUC was 0.853. Because the confidence interval for respiratory support was very wide and approached unity, this estimate should be interpreted cautiously as an exploratory signal rather than a definitive effect size.

**Table 3:** Multivariable predictors of prolonged fever (>5 days) or death before defervescence.

Predictor	Adjusted OR (95% CI)	P
Age (per 1 year)	1.24 (1.05–1.48)	<0.001
Albumin (per 1 g/dL)	0.31 (0.12–0.82)	0.003
Any respiratory support	10.00 (0.98–102.04)	0.020
Gram-negative bacteremia	5.53 (1.01–30.33)	0.021

AUC, area under the receiver operating characteristic curve; CI, confidence interval. Firth penalised-likelihood logistic regression. N = 60 episodes, 31 events. Prolonged fever is defined as time to defervescence >5 days or death before defervescence. Likelihood ratio χ²(4) = 31.2, P < 0.001. Apparent AUC = 0.879; bootstrap-optimism-corrected AUC = 0.853 (500 iterations). Confidence intervals are Wald-type while P values come from the penalised likelihood-ratio test.

Three of four predictors overlapped with the primary mortality model: any respiratory support, albumin level, and gram-negative bacteraemia. Age emerged as uniquely associated with prolonged fever independent of severity markers: each additional year of age increased the odds of prolonged fever by approximately 24%.

### Model discrimination and calibration

Both multivariable models showed high apparent discrimination and apparently preserved calibration within the derivation cohort (**Figure 3**). The primary mortality model achieved an apparent AUC of 0.912 with a 500-bootstrap optimism-corrected AUC of 0.880 (**Figure 3A**). The prolonged-fever model achieved an apparent AUC of 0.879 and a bootstrap-corrected AUC of 0.853 (**Figure 3B**). Optimism shrinkage was small for both models (0.032 and 0.026, respectively), but these internally corrected values should not be interpreted as externally validated prognostic performance. The comparable discrimination of the two models was achieved with a more favourable events-per-variable ratio for prolonged fever (31 events for 4 predictors vs 16 events for 4 predictors), implying greater inferential precision per coefficient in the prolonged-fever model. Calibration across quintiles of predicted risk was preserved for both outcomes (**Figures 3C, D**), with calibration points lying close to the line of identity. For the mortality model, the four lower-risk quintiles (n = 18, 19, 15, and 17) had observed mortality close to zero with mean predicted probabilities below 0.25, and the highest-risk quintile (n = 17) showed an observed mortality of approximately 71% against a mean predicted probability near 63%; for the prolonged-fever model, all five quintiles (n = 12 each) lay essentially on the diagonal across the full 0.08–1.00 range of predicted probability, indicating no systematic over- or under-prediction at any risk stratum.

### Exploratory competing-risk analysis of time to defervescence

As an exploratory complement to the binary prolonged-fever analysis, time to defervescence was analysed continuously with death as a competing event using Aalen-Johansen cumulative incidence and cause-specific Cox regression. Cumulative incidence of defervescence was approximately 41% by day 5 and 67% by day 10. This analysis produced directionally concordant findings but was considered supportive rather than definitive because of the small analysable sample, missing defervescence dates, and the limited number of deaths before documented defervescence.

### Additional exploratory and descriptive analyses

To further contextualise the main prognostic findings, we performed additional exploratory and descriptive analyses of respiratory-support escalation, microbiology, cohort heterogeneity, and fever-to-admission timing. Respiratory-support hierarchy analysis showed a clear escalation pattern. Among 77 episodes without respiratory support, 8 of 74 mortality-evaluable episodes died, and 22 of 50 classifiable episodes had prolonged fever. Low-flow oxygen alone was used in 4 episodes, HFNC without mechanical ventilation in 2 episodes, and mechanical ventilation in 7 episodes. Of 9 episodes in which HFNC was used, 7 subsequently required mechanical ventilation, and all 7 ventilated patients had received HFNC first. This indicates that HFNC and mechanical ventilation represented sequential points on the same respiratory-escalation pathway, explaining their similar univariable estimates and supporting the use of a composite respiratory-support severity marker in the multivariable model (**Supplementary Table 3**).

Microbiological review identified 19 positive blood cultures among 90 episodes, comprising 18 gram-negative organisms and 1 fungal isolate. Klebsiella spp., Pseudomonas aeruginosa, and Escherichia coli were the most common organisms; two isolates were multidrug-resistant E. coli. All deaths among culture-positive episodes were associated with gram-negative organisms, supporting the clinical relevance of gram-negative bacteraemia in this cohort (**Supplementary Table 4**). Descriptive stratification by diagnosis and treatment context demonstrated heterogeneity across the cohort. Mortality was highest in aplastic anaemia and AML and was more frequent in active disease than non-active disease, occurring in 9 of 23 (39%) versus 7 of 63 (11%) mortality-evaluable episodes. These subgroup findings should be interpreted descriptively only, given the small numbers within individual strata (**Supplementary Table 5**). Fever-to-admission timing was available and was summarised descriptively. Days of fever before admission had a median of 0 days (IQR 0–7; range −18 to 40). Community-onset episodes had a median of 0 days (IQR 0–8), whereas hospital-acquired episodes had negative values by definition. Fever-to-antibiotic and admission-to-antibiotic intervals were not captured in the dataset and could not be analysed.

## Discussion

In this single-centre cohort of paediatric haematology-oncology patients admitted with febrile neutropenia or sepsis during 2021-2022, adverse short-term outcomes were associated less with the depth of cytopenia itself and more with clinically accessible markers available at admission or during the early inpatient course. For in-hospital mortality, the strongest signals were requirement for respiratory support, lower admission albumin, active disease, and gram-negative bacteraemia. For prolonged fever, a related pattern emerged, with respiratory support, lower albumin, and gram-negative bacteraemia again retained, while older age replaced active disease. These findings should be interpreted as exploratory associations reflecting early or evolving illness severity rather than as proof of causal prediction.

These findings align well with current paediatric sepsis frameworks. The Phoenix consensus and the 2026 Surviving Sepsis Campaign both emphasise early organ dysfunction as the feature that distinguishes life-threatening infection from uncomplicated febrile illness (7, 8). In our analysis, respiratory support was the strongest marker of mortality and remained associated with prolonged fever, supporting the interpretation that respiratory compromise is a practical marker of evolving multi-organ dysfunction in this population. The univariable associations with tachycardia, lower oxygen saturation, lower diastolic blood pressure, oedema, high-flow nasal cannula, and mechanical ventilation reinforce the same mechanism (7, 17, 18). Importantly, high-flow nasal cannula and mechanical ventilation should not be interpreted as independent exposures in this cohort: 7 of 9 patients receiving high-flow nasal cannula were subsequently mechanically ventilated, and all 7 ventilated patients had received high-flow support first. Their similar univariable effect estimates therefore reflect sequential points on one respiratory-failure escalation pathway rather than two separate causal predictors. This supports the use of the composite “any respiratory support” in the multivariable model and the interpretation of respiratory support as a marker of early or evolving severity.

A central aim of this study was to understand outcomes across children with different underlying diagnoses. The diagnosis category showed only borderline crude association with mortality, whereas active disease status remained independently associated after adjustment. This suggests that, across mixed populations comprising predominantly acute lymphoblastic leukaemia, acute myeloid leukaemia, aplastic anaemia, and other diagnoses, current disease status may be more prognostically important than the diagnostic label alone. Children with refractory or disease-positive status are likely to have heavier prior treatment exposure, poorer marrow reserve, greater mucosal injury, and more prolonged immune dysfunction, all of which may amplify vulnerability to invasive infection and reduce resilience once sepsis develops (19–21). Contemporary paediatric FN guidance recognises diagnosis and treatment context as important modifiers of infectious risk, particularly for children with acute myeloid leukaemia, relapsed disease, prolonged neutropenia, or transplant-related immunosuppression. Our data extend that principle by suggesting that active disease captures prognostic information beyond broad diagnosis grouping at the time of hospital presentation.

Low albumin was another consistent determinant. In this setting, admission albumin is unlikely to represent nutritional status alone; rather, as a negative acute-phase protein, it may reflect the acute effects of systemic inflammation, endothelial dysfunction and trans-capillary albumin leakage, hepatic synthetic suppression, and limited physiological reserve (22, 23). Its association with both mortality and prolonged fever suggests that albumin may summarise host vulnerability more effectively than static anthropometric measures such as body mass index, which were non-informative in this cohort. From a practical perspective, albumin is promising because it is inexpensive, objective, and usually available at admission. The present findings therefore support the inclusion of albumin in early bedside risk appraisal for paediatric haemato-oncology patients presenting with FN or sepsis (22–24).

Gram-negative bacteraemia also emerged as an important determinant across both outcomes, with stronger statistical support for prolonged fever but consistent direction of effect for mortality. This is clinically plausible and concordant with current guidance on fever and neutropenia, which continues to prioritise timely empirical anti-pseudomonal coverage for high-risk children and recommends broader escalation in patients with sepsis, septic shock, resistant-organism risk, or clinical instability (2, 9). In this cohort, 19 episodes had positive blood cultures: 18 grew gram-negative organisms and 1 grew Candida krusei; no gram-positive bacteraemia was recorded. Enterobacterales and Pseudomonas aeruginosa predominated, and all deaths with a positive culture were associated with a gram-negative organism. The prolonged-fever findings are also relevant to current recommendations for reassessing persistent fever, including consideration of occult infection and invasive fungal disease when fever does not resolve despite appropriate antibacterial therapy (25, 26).

One interesting difference between the two outcomes was age. Older age did not predict mortality, but it independently increased the odds of prolonged fever. This may reflect differences in chemotherapy intensity, cumulative prior toxicity, pathogen exposure, antimicrobial resistance, or timing of presentation in older children and adolescents. However, this result should be interpreted cautiously and considered hypothesis-generating. The confidence interval was narrower than for several binary predictors because age was modelled continuously, but the biological explanation remains uncertain and deserves testing in larger multicentre cohorts. However, previous studies have documented that the older paediatric population (>16 years) and the adolescent age group are more susceptible to infection-related mortality (27). Previous studies have also documented that adolescents with cancer had a trend towards FN events and prolonged PICU stay (28).

The negative findings are also informative. Absolute neutrophil count, platelet count (<50 ×10³/µL), total leukocyte count, and IgG level did not independently predict either outcome. This should not be interpreted as evidence that cytopenia is unimportant in general; rather, it most likely reflects range restriction in a cohort already defined by severe neutropenia. Once most children are profoundly cytopenic, further subdivision of ANC may add relatively little prognostic separation in paediatric cancer/HCT FN cohorts, while broader FN data suggest that clinical course, supportive therapies, and treatment context can be more informative for hospital outcomes than static baseline cytopenia measures (29, 30). This interpretation is strengthened by the Lasso sensitivity analysis, in which no haematological variable was selected consistently. However, severe thrombocytopenia might have prognostic significance in febrile neutropenia (16, 31). In contrast, dynamic indicators of organ dysfunction and host reserve retained predictive value, suggesting that, within paediatric FN populations, bedside severity markers may be more informative than additional granularity in already-abnormal blood counts.

### Strengths, limitations and future prospects

This study has several strengths. It examined consecutive real-world admissions across multiple paediatric haemato-oncology diagnoses, used pre-specified multivariable models grounded in clinical reasoning, applied Firth penalisation to reduce small-sample bias, reported FDR-adjusted q values for multiple-testing transparency, and internally validated both models with bootstrap correction. Evaluating both mortality and prolonged fever is another strength, as these outcomes capture distinct yet complementary aspects of severe infection. Several limitations should also be acknowledged. The study was single-centred with a limited sample size; the mortality model included 16 deaths and four predictors (EPV ≈4.0), while the prolonged-fever model included 31 events and four predictors (EPV ≈7.8). Confidence intervals were wide for several estimates, and adjusted estimates whose confidence intervals included or approached unity should be interpreted as exploratory signals rather than definitive associations. Although bootstrap correction was performed, AUCs from this small derivation cohort should not be considered evidence of robust deployable prognostic performance without external validation. The prolonged-fever analysis was limited by missing defervescence dates: 30 of 90 episodes were not classifiable, including 26 discharged episodes without a documented defervescence date and 4 LAMA/DOPR episodes. Classifiable and non-classifiable episodes were similar in measured baseline variables, but selection bias cannot be excluded. The dataset did not capture exact timing of respiratory-support initiation, fever-to-antibiotic or admission-to-antibiotic intervals, vascular catheter status or catheter type, culture source (central vs peripheral), time to blood-culture positivity, full antimicrobial susceptibility profiles, or antimicrobial escalation/de-escalation timing. These omissions limit assessment of temporality, source attribution, and treatment-response pathways. Diagnosis categories and chemotherapy phases also had to be simplified, which may have reduced the ability to detect diagnosis-specific effects. IgG was unavailable in 19% of mortality-evaluable episodes, and missingness was associated with outcome; therefore, the absence of an observed IgG association should be interpreted cautiously despite consistent null findings across complete-case, median-imputed, and multiple-imputation analyses. Accordingly, the models should be regarded as clinically informative and hypothesis-generating rather than ready for direct implementation without external validation.

### Implications for future research

Our findings suggest that very low albumin may help identify a subgroup of patients who warrant closer monitoring. Whether severe hypoalbuminaemia should prompt specific supportive interventions requires prospective evaluation (24) and cannot be determined from the present observational data.

## Conclusion

Overall, these results suggest that adverse outcomes in paediatric haemato-oncology patients with febrile neutropenia are associated mainly with a combination of early or evolving organ dysfunction, underlying disease activity, host reserve, and causative microbiology, rather than by diagnosis label or the depth of cytopenia alone. At the bedside, children who require respiratory support, have low albumin, have active disease, or have gram-negative bacteraemia may represent a higher-risk subgroup, while older children with low albumin, respiratory compromise, and gram-negative bacteraemia appear more likely to experience a prolonged febrile course. These findings are consistent with current paediatric sepsis and fever-and-neutropenia guidance and support further prospective evaluation of a physiology-led, diagnosis-aware approach to early risk stratification. External validation in independent paediatric oncology cohorts is essential before any clinical deployment.

## Data Availability

The raw data supporting the conclusions of this article will be made available by the authors, without undue reservation.

## References

[1] Davis K Wilson S . Febrile neutropenia in paediatric oncology. Paediatr Child Health (Oxford). (2020) 30:93–7. doi: 10.1016/j.paed.2019.12.002 32336983 PMC7172100

[2] Lehrnbecher T Robinson PD Ammann RA Fisher B Patel P Phillips R . Guideline for the management of fever and neutropenia in pediatric patients with cancer and hematopoietic cell transplantation recipients: 2023 update. J Clin Oncol. (2023) 41:1774–85. doi: 10.1200/JCO.22.02224 36689694 PMC10022858

[3] Boeriu E Borda A Vulcanescu DD Sarbu V Arghirescu ST Ciorica O . Diagnosis and management of febrile neutropenia in pediatric oncology patients-a systematic review. Diagnostics (Basel). (2022) 12:1800. doi: 10.3390/diagnostics12081800 35892511 PMC9394251

[4] Aljabari S Balch A Larsen GY Fluchel M Workman JK . Severe sepsis-associated morbidity and mortality among critically ill children with cancer. J Pediatr Intensive Care. (2019) 8:122–9. doi: 10.1055/s-0038-1676658 31404226 PMC6687451

[5] Ali AM Sayed HA Mohammed MM . The outcome of critically ill pediatric cancer patients admitted to the pediatric intensive care unit in a tertiary university oncology center in a developing country: a 5-year experience. J Pediatr Hematol Oncol. (2016) 38:355–9. doi: 10.1097/MPH.0000000000000523 26907641

[6] Hakim H Billett AL Xu J Tang L Richardson T Winkle C . Mucosal barrier injury-associated bloodstream infections in pediatric oncology patients. Pediatr Blood Cancer. (2020) 67:e28234. doi: 10.1002/pbc.28234 32386095

[7] Schlapbach LJ Watson RS Sorce LR Argent AC Menon K Hall MW . International consensus criteria for pediatric sepsis and septic shock. JAMA. (2024) 331:665–74. doi: 10.1001/jama.2024.0179 38245889 PMC10900966

[8] Weiss SL Peters MJ Oczkowski SJW Belley-Cote E Buysse C Choong KLM . Surviving sepsis campaign international guidelines for the management of sepsis and septic shock in children 2026. Intensive Care Med. (2026) 52:937–83. doi: 10.1007/s00134-026-08360-2 41870559

[9] Lehrnbecher T Averbuch D Castagnola E Cesaro S Ammann RA Garcia-Vidal C . 8th European Conference on Infections in Leukaemia: 2020 guidelines for the use of antibiotics in paediatric patients with cancer or post-haematopoietic cell transplantation. Lancet Oncol. (2021) 22:e270–80. doi: 10.1016/S1470-2045(20)30725-7 33811814

[10] Groll AH Pana D Lanternier F Mesini A Ammann RA Averbuch D . 8th European Conference on Infections in Leukaemia: 2020 guidelines for the diagnosis, prevention, and treatment of invasive fungal diseases in paediatric patients with cancer or post-haematopoietic cell transplantation. Lancet Oncol. (2021) 22:e254–69. doi: 10.1016/S1470-2045(20)30723-3 33811813

[11] Phillips B Wade R Stewart LA Sutton AJ . Systematic review and meta-analysis of the discriminatory performance of risk prediction rules in febrile neutropaenic episodes in children and young people. Eur J Cancer. (2010) 46:2950–64. doi: 10.1016/j.ejca.2010.05.024 20621468 PMC2981857

[12] Santschi M Ammann RA Agyeman PKA Ansari M Bodmer N Brack E . Outcome prediction in pediatric fever in neutropenia: development of clinical decision rules and external validation of published rules based on data from the prospective multicenter SPOG 2015 FN definition study. PloS One. (2023) 18:e0287233. doi: 10.1371/journal.pone.0287233 37531403 PMC10395874

[13] Haeusler GM Thursky KA Slavin MA Babl FE De Abreu Lourenco R Allaway Z . Risk stratification in children with cancer and febrile neutropenia: a national, prospective, multicentre validation of nine clinical decision rules. EClinicalMedicine. (2020) 18:100220. doi: 10.1016/j.eclinm.2019.11.013 31993576 PMC6978200

[14] Phillips RS Lehrnbecher T Alexander S Sung L . Updated systematic review and meta-analysis of the performance of risk prediction rules in children and young people with febrile neutropenia. PloS One. (2012) 7:e38300. doi: 10.1371/journal.pone.0038300 22693615 PMC3365042

[15] Phillips B Morgan JE Haeusler GM Riley RD PICNICC Collaborative . Individual participant data validation of the PICNICC prediction model for febrile neutropenia. Arch Dis Child. (2020) 105:439–45. doi: 10.1136/archdischild-2019-317308 31690548 PMC7212933

[16] Das A Trehan A Bansal D . Risk factors for microbiologically-documented infections, mortality and prolonged hospital stay in children with febrile neutropenia. Indian Pediatr. (2018) 55:859–64. doi: 10.1007/s13312-018-1395-0 29941699

[17] Sanchez-Pinto LN Bennett TD DeWitt PE Russell S Rebull MN Martin B . Development and validation of the Phoenix criteria for pediatric sepsis and septic shock. JAMA. (2024) 331:675–86. doi: 10.1001/jama.2024.0196 38245897 PMC10900964

[18] Balamuth F Scott HF Weiss SL Webb M Chamberlain JM Bajaj L . Validation of the Pediatric Sequential Organ Failure Assessment Score and evaluation of third international consensus definitions for sepsis and septic shock definitions in the pediatric emergency department. JAMA Pediatr. (2022) 176:672–8. doi: 10.1001/jamapediatrics.2022.1301 35575803 PMC9112137

[19] Fisher BT Robinson PD Lehrnbecher T Steinbach WJ Zaoutis TE Phillips B . Risk factors for invasive fungal disease in pediatric cancer and hematopoietic stem cell transplantation: a systematic review. J Pediatr Infect Dis Soc. (2018) 7:191–8. doi: 10.1093/jpids/pix030 28549148 PMC12427443

[20] Martínez Campos L Pérez-Albert P Ferres Ramis L Rincón-López EM Mendoza-Palomar N Soler-Palacin P . Consensus document on the management of febrile neutropenia in paediatric haematology and oncology patients of the Spanish Society of Pediatric Infectious Diseases (SEIP) and the Spanish Society of Pediatric Hematology and Oncology (SEHOP). Pediatr (Engl Ed). (2023) 98:446–59. doi: 10.1016/j.anpede.2023.03.010 37268527

[21] Dutta A Flores R . Infection prevention in pediatric oncology and hematopoietic stem cell transplant recipients. In: Healthcare-Associated Infections in Children (2018). p. 281–99. doi: 10.1007/978-3-319-98122-2_16

[22] Dimitrijević J Čalamać M Đurmez O Krstić D Stojanović M . Serum albumin as a prognostic biomarker for febrile neutropenia outcome and complications: a prospective observational trial. Clin Med Insights Oncol. (2024) 18:11795549241281330. doi: 10.1177/11795549241281330 39323980 PMC11423384

[23] Gounden V Vashisht R Jialal I . Hypoalbuminaemia. In: Statpearls. StatPearls Publishing, Treasure Island (FL (2026). Available online at: https://www.ncbi.nlm.nih.gov/books/NBK526080/ (Accessed February 25, 2026).

[24] Cao Y Su Y Guo C He L Ding N . Albumin level is associated with short-term and long-term outcomes in sepsis patients admitted in the ICU: a large public database retrospective research. Clin Epidemiol. (2023) 15:263–73. doi: 10.2147/CLEP.S396247 36895828 PMC9990453

[25] Averbuch D Vanbiervliet Y Baccelli F Mikulska M Neofytos D Garcia-Vidal C . Empirical and targeted antimicrobial therapy in patients with febrile neutropenia and haematological Malignancy or after haematopoietic cell transplantation: recommendations from the 10th European Conference on Infections in Leukaemia. Lancet Infect Dis. (2025), S1473–3099(25)00619-X. doi: 10.1016/S1473-3099(25)00619-X 41314221

[26] Stergiotis M Ammann RA Droz S Koenig C Agyeman PKA . Pediatric fever in neutropenia with bacteraemia-pathogen distribution and *in vitro* antibiotic susceptibility patterns over time in a retrospective single-center cohort study. PloS One. (2021) 16:e0246654. doi: 10.1371/journal.pone.0246654 33577566 PMC7880464

[27] Sung L Lange BJ Gerbing RB Alonzo TA Feusner J . Microbiologically documented infections and infection-related mortality in children with acute myeloid leukemia. Blood. (2007) 110:3532–9. doi: 10.1182/blood-2007-05-091942 17660380

[28] Kirolos N Tang K Abbott LS . Does age play a role in fever and neutropenia events and complications: a comparison of adolescents versus younger children with cancer at a tertiary care pediatric hospital, a pilot project. Cancer Rep (Hoboken). (2023) 6:e1767. doi: 10.1002/cnr2.1767 36494902 PMC10075292

[29] Alali M David MZ Danziger-Isakov LA Bartlett AH Petty LA Schwartz T . Association between depth of neutropenia and clinical outcomes in febrile pediatric cancer and/or patients undergoing hematopoietic stem-cell transplantation. Pediatr Infect Dis J. (2020) 39:628–33. doi: 10.1097/INF.0000000000002641 32176187

[30] Cekin R Senocak D Cihan S . Factors influencing hospital length of stay in febrile neutropenia: a retrospective cohort study of Turkish patients with solid tumors. Med (Baltimore). (2025) 104:e43105. doi: 10.1097/MD.0000000000043105 40629594 PMC12237347

[31] Badiei Z Khalesi M Alami MH Kianifar HR Banihashem A Farhangi H . Risk factors associated with life-threatening infections in children with febrile neutropenia: a data mining approach. J Pediatr Hematol Oncol. (2011) 33:e9–e12. doi: 10.1097/MPH.0b013e3181f6921a 21102352

