# Supplementary material for: Early clinical determinants of adverse outcomes in paediatric febrile neutropenia: a prospective cohort study
Source: Front Oncol. 2026 Jul 29;16:1876009. doi: 10.3389/fonc.2026.1876009 (PMC13461490; doi:10.3389/fonc.2026.1876009)
Supplement: Supplementary file 1 [file DataSheet1.pdf]

## Early Clinical Determinants of Adverse Outcomes in Paediatric Febrile Neutropenia: A Prospective Cohort Study

Manideepa Maji; Nita Radhakrishnan; Shruti Verma; Aishvarya Diptivilasa; Saikat Mandal

**Supplementary Table S1:** Univariable Firth-penalised likelihood logistic regression estimates for all candidate predictors against in-hospital mortality and prolonged fever.

In-hospital mortality cohort: n = 86, 16 deaths.    Prolonged-fever cohort: n = 60, 31 events.

| **Predictor** | **In-hospital mortality** | | **Prolonged fever** | |
|---|---|---|---|---|
| | **OR (95% CI)** | **P** | **OR (95% CI)** | **P** |
| ***Demographics*** — Age (per 1 year) | 1.08 (0.95–1.23) | 0.220 | 1.22 (1.06–1.41) | 0.002 |
| Male sex | 0.33 (0.11–0.99) | 0.049 | 0.52 (0.17–1.58) | 0.224 |
| BMI (per 1 kg/m²) | 1.09 (0.88–1.35) | 0.488 | 0.96 (0.78–1.18) | 0.660 |
| ***Disease*** — Active disease | 4.94 (1.59–15.35) | 0.005 | 2.32 (0.74–7.30) | 0.126 |
| Relapsed/refractory diagnosis | 1.68 (0.41–6.85) | 0.567 | 0.75 (0.20–2.79) | 0.649 |
| Intensive-phase chemotherapy | 1.84 (0.48–7.02) | 0.315 | 1.70 (0.54–5.31) | 0.335 |
| Community-onset FN | 0.69 (0.14–3.44) | 0.834 | 1.08 (0.20–5.81) | 0.931 |
| ***Vitals*** — Heart rate (per 1 bpm) | 1.06 (1.03–1.10) | <0.001 | 1.01 (0.98–1.03) | 0.461 |
| Respiratory rate (per 1 breath/min) | 1.05 (0.99–1.11) | 0.038 | 1.02 (0.96–1.08) | 0.397 |
| SBP (per 1 mmHg) | 0.94 (0.89–0.99) | 0.010 | 0.97 (0.93–1.02) | 0.171 |
| DBP (per 1 mmHg) | 0.91 (0.86–0.97) | <0.001 | 0.96 (0.92–1.01) | 0.059 |
| Age-adjusted hypotension | 3.83 (0.77–19.08) | 0.120 | 3.94 (0.52–29.78) | 0.092 |
| $SpO_2$ (per 1%) | 0.62 (0.44–0.88) | <0.001 | 0.81 (0.61–1.09) | 0.042 |
| GCS <15 | 2.65 (0.24–29.30) | 0.548 | 0.93 (0.06–15.67) | 0.962 |
| Urine output (per 1 mL/kg/hr) | 0.72 (0.47–1.09) | 0.102 | 1.00 (0.69–1.46) | 0.982 |
| ***Respiratory*** — Supplemental $O_2$ | 14.78 (3.68–59.31) | <0.001 | 8.02 (1.21–53.12) | 0.005 |
| High-flow nasal cannula | 36.58 (5.05–265.1) | <0.001 | 18.06 (0.81–404.1) | 0.002 |
| Mechanical ventilation | 111.3 (4.84–2560) | <0.001 | 18.06 (0.81–404.1) | 0.002 |
| Any respiratory support | 14.78 (3.68–59.31) | <0.001 | 8.02 (1.21–53.12) | 0.005 |
| ***Supportive*** — Fluid resuscitation | 6.94 (1.38–34.81) | 0.016 | 1.80 (0.31–10.39) | 0.439 |
| Significant oedema | 27.40 (5.29–141.8) | <0.001 | 28.81 (1.39–595.2) | <0.001 |
| ***Haematology*** — TLC (per 1000/µL) | 1.00 (0.56–1.79) | 1.000 | 0.89 (0.51–1.55) | 0.655 |
| ANC (per 100/µL) | 0.99 (0.81–1.20) | 0.865 | 1.00 (0.84–1.19) | 0.960 |
| ANC <1000/µL | 0.22 (0.01–3.77) | 0.309 | 0.20 (0.005–8.54) | 0.160 |

| | | | | |
|---|---|---|---|---|
| ANC <500/µL | 0.79 (0.16–3.86) | 1.000 | 0.64 (0.14–2.92) | 0.511 |
| ANC <100/µL | 1.36 (0.46–4.08) | 0.562 | 1.92 (0.67–5.46) | 0.205 |
| Platelets (per 10 × $10^3/\mu L$) | 1.00 (0.79–1.26) | 1.000 | 0.94 (0.77–1.16) | 0.572 |
| Platelets <50 × $10^3/\mu L$ | 0.75 (0.19–2.95) | 0.815 | 1.33 (0.36–4.95) | 0.649 |
| Platelets <10 × $10^3/\mu L$ (post hoc) | 3.12 (1.04–9.38) | 0.040 | 2.03 (0.71–5.82) | 0.169 |
| ***Biochemistry* —** Creatinine (per 1 mg/dL) | 2.78 (0.48–16.04) | 0.204 | 0.87 (0.16–4.72) | 0.827 |
| Age-adjusted AKI | 4.72 (0.61–36.38) | 0.144 | 0.93 (0.12–7.09) | 0.945 |
| Albumin (per 1 g/dL) | 0.23 (0.09–0.55) | <0.001 | 0.36 (0.16–0.80) | 0.004 |
| Hypoalbuminaemia (<3.5 g/dL) | 4.57 (1.27–16.43) | 0.008 | 2.50 (0.88–7.07) | 0.072 |
| Sodium (per 1 mmol/L) | 1.03 (0.95–1.11) | 0.553 | 1.02 (0.94–1.11) | 0.430 |
| Potassium (per 1 mmol/L) | 0.71 (0.32–1.61) | 0.416 | 0.70 (0.33–1.47) | 0.310 |
| Random blood glucose (per 10 mg/dL) | 0.87 (0.71–1.06) | 0.133 | 0.99 (0.84–1.17) | 0.905 |
| IgG (per 100 mg/dL)[1] | 0.86 (0.63–1.19) | 0.244 | 1.05 (0.86–1.28) | 0.632 |
| ***Microbiology* —** Any positive blood culture | 2.90 (0.89–9.40) | 0.088 | 6.17 (1.33–28.57) | 0.005 |
| Gram-negative bacteraemia | 3.20 (0.97–10.53) | 0.063 | 6.17 (1.33–28.57) | 0.005 |
| ***Prior exposures* —** Prior corticosteroid | 1.21 (0.31–4.73) | 1.000 | 0.64 (0.19–2.15) | 0.452 |

[1] Serum IgG was measured in 70/86 (81.4%) of episodes in the mortality cohort and 50/60 (83.3%) of episodes in the prolonged-fever cohort. Missingness was strongly associated with both outcomes (P < 0.001 for mortality; P = 0.013 for prolonged fever), indicating an informative-missingness pattern most likely arising from clinical workflow: severely ill patients who deteriorated before laboratory work-up was completed have IgG values systematically missing. Estimates are from complete-case analysis and may be biased if missingness was informative; IgG was therefore not retained in any multivariable model.

**Abbreviations:** AKI, acute kidney injury; ANC, absolute neutrophil count; BMI, body mass index; CI, confidence interval; DBP, diastolic blood pressure; FN, febrile neutropenia; GCS, Glasgow Coma Scale; HFNC, high-flow nasal cannula; IgG, immunoglobulin G; OR, odds ratio; RBS, random blood sugar; SBP, systolic blood pressure; $SpO_2$, peripheral oxygen saturation; TLC, total leukocyte count.

All estimates derived from Firth penalised-likelihood logistic regression. Each predictor was fitted independently against each outcome. Continuous predictors are reported per the unit shown in parentheses; binary predictors compare presence vs absence. P values are derived from the likelihood-ratio test of the Firth-penalised model; confidence intervals are Wald-type. P values are two-sided and uncorrected for multiple testing. The full predictor screen is reported for transparency, while the multivariable models reported in the main text were pre-specified on clinical grounds rather than selected by univariable significance. Severe thrombocytopenia (<10 × $10^3/\mu L$) was added to the screen as a post hoc exploratory threshold.

## Additional Supplementary Tables

### Supplementary Table S1a: Benjamini-Hochberg FDR q values for univariable screens.

| Predictor | Mortality q | Prolonged fever q |
|---|---|---|
| Age (per 1 year) | 0.373 | 0.023 |
| Male sex | 0.128 | 0.485 |
| BMI (per 1 kg/m²) | 0.680 | 0.804 |

| | | |
|---|---|---|
| Active disease | 0.020 | 0.350 |
| Relapsed/refractory diagnosis | 0.691 | 0.804 |
| Intensive-phase chemotherapy | 0.472 | 0.654 |
| Community-onset FN | 0.956 | 0.982 |
| Heart rate (per 1 bpm) | <0.001 | 0.719 |
| Respiratory rate (per 1 breath/min) | 0.107 | 0.719 |
| SBP (per 1 mmHg) | 0.034 | 0.418 |
| DBP (per 1 mmHg) | 0.001 | 0.209 |
| Age-adjusted hypotension | 0.246 | 0.277 |
| $SpO_2$ (per 1%) | <0.001 | 0.164 |
| GCS <15 | 0.691 | 0.982 |
| Urine output (per 1 mL/kg/hr) | 0.220 | 0.982 |
| Supplemental $O_2$ | <0.001 | 0.023 |
| High-flow nasal cannula | <0.001 | 0.023 |
| Mechanical ventilation | <0.001 | 0.023 |
| Any respiratory support | <0.001 | 0.023 |
| Fluid resuscitation | 0.049 | 0.719 |
| Significant oedema | <0.001 | 0.007 |
| TLC (per 1000/µL) | 1.000 | 0.804 |
| ANC (per 100/µL) | 0.964 | 0.982 |
| ANC <1000/µL | 0.472 | 0.415 |
| ANC <500/µL | 1.000 | 0.766 |
| ANC <100/µL | 0.691 | 0.470 |
| Platelets (per $10\times10^3$/µL) | 1.000 | 0.804 |
| Platelets <$50\times10^3$/µL | 0.956 | 0.804 |
| Creatinine (per 1 mg/dL) | 0.361 | 0.978 |
| Age-adjusted AKI | 0.268 | 0.982 |
| Albumin (per 1 g/dL) | <0.001 | 0.023 |
| Hypoalbuminaemia (<3.5 g/dL) | 0.029 | 0.233 |
| Sodium (per 1 mmol/L) | 0.691 | 0.719 |
| Potassium (per 1 mmol/L) | 0.601 | 0.636 |
| Random blood glucose (per 10 mg/dL) | 0.259 | 0.982 |
| IgG (per 100 mg/dL) | 0.396 | 0.804 |
| Any positive blood culture | 0.201 | 0.023 |
| Gram-negative bacteraemia | 0.155 | 0.023 |
| Prior corticosteroid | 1.000 | 0.719 |
| Platelets <$10\times10^3$/µL (post hoc) | — | — |

Benjamini-Hochberg correction was applied separately for each outcome across the 39 a priori predictors. The post hoc platelet threshold (<$10\times10^3$/µL) was displayed but excluded from the FDR family.

**Supplementary Table S2: Classifiable vs non-classifiable episodes for prolonged fever**

| Baseline variable | Classifiable (n=60) | Non-classifiable (n=30) | P |
|---|---|---|---|
| Age, y | 8.0 [4.8–12.0] | 8.0 [5.0–11.8] | 0.800 |
| BMI, kg/m² | 14.6 [13.7–15.7] | 14.6 [13.9–15.7] | 0.669 |
| Albumin, g/dL | 3.4 [2.8–3.8] | 3.5 [2.8–3.8] | 0.706 |
| Heart rate, bpm | 132.0 [114.0–145.0] | 132.0 [112.0–143.0] | 0.560 |
| Days to discharge | 7.0 [5.5–10.0] | 9.5 [6.3–10.0] | 0.138 |
| Active disease | 18/60 (30%) | 8/30 (27%) | 0.809 |
| Any respiratory support | 10/60 (17%) | 3/30 (10%) | 0.532 |
| Culture positive | 13/60 (22%) | 6/30 (20%) | 1.000 |
| Gram-negative bacteraemia | 13/60 (22%) | 5/30 (17%) | 0.781 |
| Intensive chemo | 38/56 (68%) | 14/27 (52%) | 0.226 |

Observed prolonged-fever prevalence was 31/60 (51.7%). If all 30 non-classifiable episodes were not prolonged, prevalence would be 31/90 (34.4%); if all were prolonged, prevalence would be 61/90 (67.8%).

**Supplementary Table S3: Outcomes by respiratory-support level**

| Respiratory-support level | n | In-hospital mortality | Prolonged fever |
|---|---|---|---|
| No respiratory support | 77 | 8/74 (11%) | 22/50 (44%) |
| Low-flow oxygen only | 4 | 1/4 (25%) | 2/3 (67%) |
| HFNC without mechanical ventilation | 2 | 0/1 (0%) | — |
| Mechanical ventilation | 7 | 7/7 (100%) | 7/7 (100%) |

Of 9 patients who received high-flow nasal cannula, 7 (78%) were subsequently mechanically ventilated, and all 7 ventilated patients had received HFNC first.

**Supplementary Table S4: Blood-culture organisms and associated outcomes**

| Organism | n | Deaths | Prolonged fever |
|---|---|---|---|
| Klebsiella spp. | 4 | 2/4 | 2/2 |
| Pseudomonas aeruginosa | 4 | 1/3 | 3/3 |
| Escherichia coli | 3 | 0/3 | 2/3 |
| Escherichia coli (MDR) | 2 | 2/2 | 2/2 |
| Acinetobacter lwoffii | 1 | 0/1 | 1/1 |
| Candida krusei | 1 | 0/1 | — |
| Enterobacter cloacae | 1 | 0/1 | — |
| Moraxella spp. | 1 | 1/1 | 1/1 |

| | | | |
|---|---|---|---|
| Ochrobactrum anthropi | 1 | 0/1 | 0/1 |
| Vibrio cholerae | 1 | 0/1 | — |

Denominators reflect classifiable episodes for each outcome.

**Supplementary Table S5: Outcomes stratified by diagnosis and treatment characteristics**

| **Diagnosis category** | **n** | **Deaths** | **Prolonged fever** |
|---|---|---|---|
| ALL / lymphoid leukaemia | 61 | 8/60 (13%) | 20/43 (47%) |
| AML | 12 | 3/10 (30%) | 5/8 (63%) |
| Lymphoma (NHL/Burkitt/ALCL) | 4 | 1/4 (25%) | 2/4 (50%) |
| Aplastic anaemia | 8 | 4/7 (57%) | 4/4 (100%) |
| Solid tumour | 4 | 0/4 (0%) | 0/1 (0%) |
| LCH | 1 | 0/1 (0%) | — |
| **Stratum** | **n** | **Deaths** | **Prolonged fever** |
| Active disease | 26 | 9/23 (39%) | 12/18 (67%) |
| Non-active disease | 64 | 7/63 (11%) | 19/42 (45%) |
| Intensive-phase chemo | 52 | 9/49 (18%) | 20/38 (53%) |
| Non-intensive chemo | 31 | 3/30 (10%) | 7/18 (39%) |
| Community-onset FN | 81 | 14/77 (18%) | 28/54 (52%) |
| Hospital-acquired FN | 9 | 2/9 (22%) | 3/6 (50%) |

Subgroup estimates are descriptive only because of small per-stratum counts.

**Supplementary Table S6: One-episode-per-patient sensitivity analysis for mortality**

| **Predictor** | **Adjusted OR (95% CI)** | **P** |
|---|---|---|
| Any respiratory support | 12.25 (2.31–64.87) | <0.001 |
| Albumin (per 1 g/dL) | 0.30 (0.11–0.79) | 0.005 |
| Active disease | 2.97 (0.69–12.85) | 0.143 |
| Gram-negative bacteraemia | 4.52 (0.92–22.25) | 0.050 |

Firth penalised likelihood model, n=74 episodes, 16 deaths. Model $\chi^2(4)=32.6$, $P<0.001$; apparent AUC 0.914; bootstrap-optimism-corrected AUC 0.884 (500 resamples).
